# Ultra-Stretchable Polymer Fibers Anchored with a Triple-Level Self-Assembled Conductive Network for Wide-Range Strain Detection

**DOI:** 10.3390/polym17060734

**Published:** 2025-03-11

**Authors:** Zhong Zheng, Shuyi Song, Xun Chen, Xixing Li, Jing Li

**Affiliations:** 1 Hubei Key Laboratory of Modern Manufacturing Quantity Engineering, School of Mechanical Engineering, Hubei University of Technology, Wuhan 430068, China; songshuyi1016@163.com (S.S.); cx747699@163.com (X.C.); li_xi_xing@126.com (X.L.); 2 School of Intelligent Manufacturing, Hubei University, Wuhan 430062, China

**Keywords:** buckling, TPU microfibers, conductive network, stretchable sensor

## Abstract

Numerous strategies have been demonstrated to enhance the mechanical stretchability of electromechanical sensors for widespread applications in wearable electronics. However, ranging from composite to microstructural materials, their electromechanical sensing performances are usually vulnerable to large stretching deformations due to the low-ductility of the infilled conductive components and the modulus mismatch between the flexible polymer substrate and conductive fillers. Here, a novel design strategy is proposed to fabricate ultra-stretchable electromechanical composites constructed by a triple-level interaction conductive network (Tri-LICN) in buckled-TPU microfibers for strain sensors. The Tri-LICN is established by bridging one-dimensional cellulose nanocrystals (CNC) with zero-dimensional gold-nanoparticles (AuNPs) and two-dimensional MXene sheets using interface self-assembly and ultrasound-assisted anchoring to eliminate the modulus mismatching between the conductive material and polymer substrate. The buckled-TPU microfibers are introduced to improve the mechanical stretchability of composites by the external-stimuli-induced imbalance of the stretching conformation of TPU macromolecules. The Tri-LICN MXene/CNC/AuNPs@TPU composite sensor displays an enhanced strain sensitivity (GF~2514) with a fast response time (~150 ms) over a wide operational strain up to 200% and excellent durability over 1000 tensile cycles. Our finding offers a promising approach to enhancing the performance of stretchable sensors based on polymer materials, providing new opportunities for the development of next-generation electronics.

## 1. Introduction

Stretchable electromechanical materials have become one of the most attractive components of wearable electronic devices for measuring and quantifying human physical stimuli [1]. However, the strain extents across different parts of the human body vary significantly, spanning two orders of magnitude. For example, the strains at the joints of the limbs can be as high as 100%, while the strain on the epidermis caused by the vibration of the laryngeal cartilage during vocalization is less than 5% [2]. Thus, there is an urgent need to develop stretchable strain sensors with durable and stable electromechanical performances across a wide strain range to monitor human large-strain behaviors [3,4]. Researchers have made substantial efforts in the past decades to address this issue, with resistive stretchable thin-film strain sensors generally consisting of flexible substrates and conductive materials [4]. Commonly used conductive materials include highly conductive metal nanoparticles, such as gold nanoparticles (AuNPs) and silver nanoparticles (AgNPs), as well as carbon black (CB), all of which are zero-dimensional (0D) materials. Still, their inherent structural brittleness leads to discontinuous conductive layers under small strains, limiting their strain range [5]. One-dimensional (1D) materials, such as carbon nanotubes (CNTs) and silver nanowires, with high aspect ratios, can be combined with 0D materials to extend the tensile range of the conductive layer. However, 1D materials often exhibit poor tensile and bending properties, making them prone to fracture or contact failures [6,7].

To overcome the limited sensing performances of low-dimensional materials under large tensile strains, the excellent ductility of two-dimensional (2D) conductive materials, such as graphene oxide (GO), black phosphorus (BP), and MXene, is utilized to optimize the tensile range of stretchable strain sensors [8,9,10]. MXene demonstrates exceptional metallic conductivity (2.4 × 10^5^ S·cm^−1^) and biocompatibility, among other advantages [11]. Since its first synthesis by Gogotsi et al. in 2011 [12], it has become a prominent candidate for conductive materials in flexible strain sensors [12,13]. For instance, a stretchable sensor exhibiting an 80% strain range was prepared using an impregnation–drying method in which MXene was encapsulated on an electrospun thermoplastic polyurethane/polyacrylonitrile (TPU/PAN) fiber membrane [14]. In another approach, MXene was vacuum-filtered onto an electrospun TPU fiber membrane and assembled with a TPU/boron nitride nanosheet composite membrane, resulting in a stretchable sandwich-like strain sensor with a sensitivity of up to 2080.9 and a strain operating range of 100% [13]. However, the abundant chemical functional groups (-O, -OH, -F) on MXene nanosheets lead to strong interlayer interactions, which facilitate dense stacking of adjacent nanosheets, resulting in uneven loading [15]. Under large strains, crack propagation becomes the dominant sensing mechanism rather than sliding, as cracks cause the conductive pathways to disconnect prematurely, thereby limiting the extensibility of the conductive network. These factors constrain the sensing range of strain sensors and hinder their ability to meet the long-term cycling requirements of the devices [16].

To address the dense stacking issue of 2D MXene sheets, researchers have investigated the freeze-casting technique, which dissociates the stacked MXene sheets through the volume expansion of water within the interlayer spaces [14,15]. However, this method still faces the challenge that MXene tends to peel off under large stretching, affecting the stability required for stretchable sensors [17]. Therefore, further improvements in the stability and ductility of MXene dispersions are necessary. Our strategy involves introducing conductive materials with different dimensionalities. Firstly, MXene and cellulose nanocrystal (CNC) composites are promising candidates for stretchable strain sensors [18,19]. The 1D material CNC is composed of repeating β-(1,4)-linked D-glucan units with the molecular formula (C_6_H_10_O_5_)_n_ [16]. The material exhibits a Young’s modulus ranging from 20 to 50 GPa and offers several advantages, including biocompatibility, low cost, and scalability for large-scale production [20]. CNC is among the most abundant renewable biopolymers on earth [21]. More importantly, the surface of CNC is rich in functional groups (-OH), which can form hydrogen bonds with the chemical end groups (-OH, -F) on the surface of MXene, thereby connecting the interlayer nanospaces of adjacent 2D MXene nanosheets [22]. By combining CNC with MXene, electrostatic interactions can stabilize the dispersion of MXene in water, thereby preventing the aggregation of MXene nanosheets [23]. Hydrogen bonding can effectively enhance the adhesion of MXene to the TPU substrate [24]. The CNC-bridged MXene sheets facilitate orderly sliding between the sheets, preventing premature rupture of the conductive path and enhancing the sensing range. However, CNC is an electrical insulator. This presents a challenge: the electron transfer between MXene sheets may be impeded by the presence of the insulating CNC, ultimately reducing the electrical performance and sensitivity of the sensing network. Gold nanoparticles (AuNPs), classified as a 0D material, exhibit outstanding electrical conductivity, chemical inertness, biocompatibility, and antimicrobial properties, making them an ideal conductive nanomaterial for designing wearable sensors [25]. The integration of AuNPs and MXene also enhances the electro-thermal and photo-thermal effects of the composites, potentially facilitating practical applications in health monitoring, healthcare, and sports monitoring [26,27]. Therefore, incorporating highly conductive 0D AuNPs into the conductive network sensing layer establishes rapid charge transfer channels, thereby enhancing both the sensitivity and response speed of the flexible strain sensor. In conclusion, the synergistic sensing network formed by MXene/CNC/AuNP composites not only promotes the sliding and bridging of MXene sheets but also ensures the uniform distribution of sensing materials while enriching the conductive pathways.

However, improving sensor performance solely by enhancing the conductive materials is inherently limited. Consequently, a key challenge lies in fabricating flexible polymer film substrates with enhanced deformability and distinctive microstructures through simple and effective methods. Recently, considerable efforts have concentrated on designing unique microstructures to broaden the strain detection range of stretchable sensors [28]. The researchers have investigated an array of sensing mechanisms, including crack gradient propagation [29], microstructures (such as folding, buckling, and springing) [30,31], conductive material slip [32], polymorphic conductive networks [33], and geometric transformations [34] to induce gradients in microcracks or extend multiple conductive networks under heightened tensile strain. Among these methods, enhancing the structural deformation redundancy of stretchable polymer thin-film substrates has been identified as an effective strategy for improving the tensile deformation capability of stretchable strain sensors. For example, stretchable strain sensors can detect strains of up to 100% by creating a parallel through-hole array structure on a flexible conductive polymer film using laser cutting, achieving a maximum sensitivity of 657.36 [28]. Additionally, a two-component off-axis electrospinning method has been employed to deform nanofibers asymmetrically, and ultra-stretched ceramic aerogels with high buckling structures have been fabricated, enabling tensile strain up to 150% [35]. However, improving the stretchability of strain sensors frequently presents additional challenges. Repeated large mechanical strains can cause irreversible fractures, resulting in substantial degradation of the conductive pathways [19]. Moreover, the insufficient bonding between MXene and the stretchable polymer substrate and a significant modulus mismatch lead to MXene delaminating or undergoing irreversible migration under large strains [4,6]. This results in the deterioration of sensing performance and impedes the achievement of durability and long-term stability in flexible sensors. Therefore, an efficient, low-cost, and simple strategy to integrate flexible polymer substrates with a conductive network that ensures modulus compatibility is urgently needed to fabricate stretchable strain sensors with a wide strain range.

**Figure 1:**
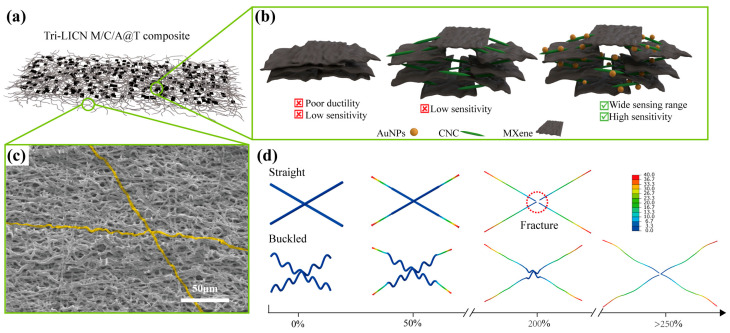
Structural design of the stretchable Tri-LICN M/C/A@T composite. (**a**) Structural schematic of the Tri-LICN M/C/A@T composite. (**b**) The detailed microstructure of Tri-LICN comprising 2D MXene, 1D CNC, and 0D AuNPs. (**c**) SEM image of the buckled-TPU microfiber. The magnification is ×500. (**d**) FEA simulated illustrations of the enhanced stretchability through buckling microstructure.

In this study, an ultra-stretchable composite strain sensor was developed using highly buckled TPU microfibers with a triple-level interaction conductive network (Tri-LICN) for wide-range detection. The superior stretchability of the composite was achieved by Tri-LICN in buckled-TPU microfibers fabricated through a hot solvent swelling process and ultrasound-assisted anchoring technology. As shown in Figure 1a, the 0D/1D/2D (AuNPs/CNC/MXene) triple-level nanocomposite active sensing elements are uniformly distributed on a buckled-TPU microfiber. The synergistic effect of the buckled TPU microstructure and the rich interfacial interactions endows the Tri-LICN M/C/A@T composite sensor with exceptional large-strain electromechanical response characteristics, including a wide detection range (0.05–200%), high sensitivity (~2514), fast response time (~150 ms), rapid recovery time (~200 ms), and remarkable mechanical stress resistance over 1000 cycles of stretch/release loadings. These outstanding response characteristics permit the as-proposed composite sensor to accommodate various body types and detect a wide range of movements, from limb motions to subtle actions such as facial expressions and swallowing. The Tri-LICN M/C/A@T composite sensor can accurately and stably monitor the large tensile strain behaviors of the human body. Moreover, theoretical modeling and finite element simulations were employed to elucidate the deformation mechanisms of the buckled structures of the stretchable polymer substrate, with results showing good agreement with experimental observations. By integrating the benefits of flexible substrates with distinctive microstructures and conductive networks made from multidimensional materials, this approach offers a novel method for designing and fabricating advanced sensors based on polymers that exhibit rapid responses under large tensile strains.

## 2. Materials and Methods

### 2.1. Materials

Ti_3_AlC_2_ (MAX) was purchased from Jilin Yiyi Technology Co., Ltd., Changchun, China; thermoplastic polyurethane TPU (1185A, Tg = −38 °C) was purchased from BASF Chemical (Shanghai, China); chloroauric acid (HAuCl_4_), sodium citrate (Na_3_C_6_H_5_O_7_·2H_2_O), N,N-dimethylformamide (DMF), tetrahydrofuran (THF), lithium fluoride (LiF), hydrochloric acid (HCl, 98 wt%), sulfuric acid (H_2_SO_4_, 98 wt%), and ethanol (C_2_H_5_OH) were purchased from Sinopharm Chemical Reagent Co., Ltd. (Shanghai, China); medical degreasing cotton balls were purchased from Kangji Pharmaceutical Machinery Co., Ltd. (Shijiazhuang, China); polyimide (PI) tape was purchased from Ming Shen Electronic Technology Development Co., Ltd. (Shanghai, China); deionized (DI) water was used in all experiments, and all solvents were of analytical grade which could be used without further purification.

### 2.2. Preparation of MXene Suspension

LiF (2 g) was dissolved in HCl (40 mL, 9 M) and stirred for 30 min in a polytetrafluoroethylene (PTFE) vessel. Subsequently, MAX (2 g) was slowly added to the solution, and the mixture was stirred continuously for 24 h at 35 °C (400 rpm). The reaction product was centrifuged at 3500 rpm for 10 min. The supernatant was discarded, and 20 mL of DI water was added to the precipitate and mixed thoroughly. This washing step was repeated until the pH of the solution reached or exceeded 6. The washed precipitate was then dispersed in 40 mL of DI water and ultrasonicated in an ice-water bath for 1 h. After ultrasonication, the solution was again centrifuged at 3500 rpm for 30 min to obtain the supernatant, which contained the exfoliated MXene suspension. The concentration of the MXene suspension was determined to be 26 mg/mL by vacuum filtration.

### 2.3. Preparation of CNC Suspension

The CNC suspension was prepared using medical cotton as the raw material through H_2_SO_4_ hydrolysis. Firstly, medical cotton (4 g) was added to 70 mL of H_2_SO_4_ solution (64 wt%) and stirred at 400 rpm under a water bath at 45 °C for 1 h. The hydrolysis was immediately stopped by diluting the suspension with DI water to more than 10 times the initial volume. The suspension was centrifuged repeatedly at 10,000 rpm for 10 min, 2–3 times. The resulting suspension was dialyzed using a pretreated dialysis bag (MWCO: 1000 Da) until the pH reached neutral. The suspension after dialysis was sonicated in an ice-water bath for 30 min to ensure uniform dispersion. Finally, the obtained CNC suspension was concentrated to 6.5 mg/mL by rotary evaporation.

### 2.4. Preparation of AuNP Suspension

The gold nanoparticle (AuNP) solution was prepared by the reduction method using chloroauric acid (HAuCl_4_) and sodium citrate (Na_3_C_6_H_5_O_7_·2H_2_O) as precursors. Firstly, DI water (100 mL) was heated to 100 °C, and 2 mL of HAuCl_4_ (1 wt%) was quickly added to the boiling water while stirring slowly at 300 rpm. Subsequently, sodium citrate solution (6 mL, 1 wt%) was rapidly introduced into the boiling mixture, and the stirring speed was increased to 1000 rpm. The solution color gradually changed from yellow to blue to deep red. The mixture was then stirred continuously at 100 °C for 10 min. The resulting AuNP suspension was allowed to cool to room temperature and stored in a refrigerator for further use.

### 2.5. Preparation of TPU Fiber Membrane Flexible Substrates

Preparation of a TPU fiber membrane by the electrospinning method: Firstly, the TPU particles were dried in a vacuum oven at 60 °C for 24 h to remove any moisture. Then, TPU (10 wt%) particles were dissolved in 20 mL of DMF/THF mixed solution (V_DMF_: V_THF_ = 1:1). The mixture was heated in a water bath at 60 °C and magnetically stirred (500 rpm) until the TPU particles were fully dissolved. The electrospinning parameters were set as follows: the needle size was 19, the voltage was set to 20 kV, the extrusion speed was 1 mL/h, and the distance between the syringe needle and the metal drum receiver was 15 cm. The rotation speeds of the metal drum receiver were set to 200, 500, and 1000 r/min. Finally, TPU fiber membranes with dimensions of 20 cm × 10 cm were obtained.

Hot solvent swelling process for building buckled structures: The previously prepared TPU fiber membrane was left at room temperature for 24 h. Then, the ethanol solution was placed in a vacuum oven and heated to 60 °C. The TPU fiber membrane was immersed in the ethanol solution and maintained at 60 °C for 1 h. Afterward, the TPU fiber membrane was removed from the solution and left at room temperature until the ethanol was completely evaporated, resulting in TPU fiber membranes with a buckled structure. The TPU fiber membranes were then cut into pieces measuring 3 cm × 1 cm for further use.

### 2.6. Preparation of Stretchable Strain Sensors

**Figure 2:**
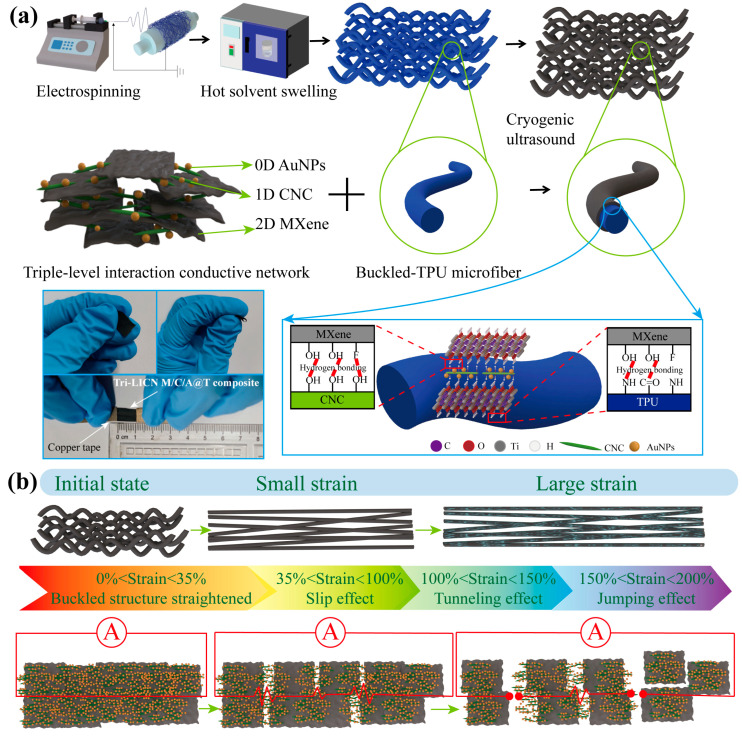
Schematic fabrication and electromechanical sensing mechanism of Tri-LICN M/C/A@T composite. (**a**) Fabrication processes of buckled-TPU microfibers and hydrogen bonding via electrostatic self-assembly strategies for formation of 2D MXene/1D CNC/0D AuNPs Tri-LICN. (**b**) Illustrated electromechanical sensing mechanism for Tri-LICN M/C/A@T composite during different mechanical stretching ranges.

Preparation of Tri-LICN M/C/A@T composite: The preparation process is illustrated in Figure 2a. AuNP suspension (1.5 mL) was added to 6 mL of CNC suspension and sonicated (480 W) for 3 min. Then, MXene suspension (1 mL) was introduced into the mixture, followed by sonication for 10 min, resulting in an MXene/CNC/AuNP suspension with an MXene concentration of 3 mg/mL, where the mass ratio of MXene to CNC was 1:1.5. Control group solutions were prepared with MXene to CNC mass ratios of 1:1.0 and 1:0.5. Next, TPU fiber membranes with a buckled structure, prepared in Section 2.5, were immersed in the MXene/CNC/AuNP suspension and sonicated for 30 min. The M/C/A@T composite fiber membranes were removed from the suspension and washed three times with DI water to remove any unanchored conductive materials. The M/C/A@T composite fiber membranes were dried at room temperature for 24 h, yielding the Tri-LICN M/C/A@T composites. The TPU fiber membranes without a hot solvent swelling treatment were denoted as “un-T”. TPU fiber membranes obtained from the electrospinning process with different metal drum receiver rotation speeds are denoted as “Tx”, where x represents the rotation speed of the metal drum receiver, such as T200, T500, and T1000. The MXene to CNC mass ratios of 1:1.5, 1:1.0, and 1:0.5 are denoted as M/C1.5, M/C1.0, and M/C0.5, respectively.

Assembly of Tri-LICN M/C/A@T composite sensor: Conductive copper tape was used as electrodes, with the adhesive side fixed to both ends of the Tri-LICN M/C/A@T composite. Wires were then connected to both ends to complete the fabrication of the Tri-LICN M/C/A@T composite sensor. In all applications, the Tri-LICN M/C1.5/A@T1000 composite sensor was employed. The stretchable strain sensor is composed of MXene/CNC/AuNPs with the mass ratio of MXene to CNC of 1:1.5 as the conductive material, which is anchored and distributed on TPU fiber. The TPU fiber film is obtained by hot solvent swelling after electrostatic spinning with the rotating speed of the metal drum receiver of 1000 r/min.

### 2.7. Characterization

The surface morphology of the sensors under different strains was observed using a scanning electron microscope (SEM, Zeiss GeminiSEM 500, Carl Zeiss AG, Oberkochen, Germany) and a homemade sample holder. The elemental content and distribution of the conductive fillers on the TPU films were investigated in detail using its accompanying energy spectrometer (EDS). The electromechanical properties of the prepared sensors were analyzed using a universal testing machine (CMT6103, Metersbonwe industrial system Co., Ltd, Shanghai, China), while the resistance changes at a constant voltage of 1 V were recorded in real time on a digital bridge (VC4092A, Shenzhen Yisheng Shengli technology Co., Ltd, Shenzhen, China). The electromechanical properties of the M/C/A@T composite film were tested and analyzed through tensile testing to evaluate its mechanical performance, stress–strain behavior, and electrical response. The sample, in the form of a rectangular film, had dimensions of 1 cm × 3 cm, and the tensile test was conducted along the 3 cm length direction. Both ends of the sample were secured in the tensile grips of a universal testing machine, ensuring uniform alignment and gripping of the material. The edges of the sample were carefully trimmed to ensure neatness, with no visible cracks or defects. Electrodes of a digital bridge were connected to the leads extending from both ends of the sample, allowing for current to flow through the composite material for resistance measurement. The tensile test was performed according to the ASTM D882 standard. The experiment was carried out at room temperature (23 °C ± 1 °C), in a dry environment with a humidity level controlled at 50% ± 2% to avoid performance changes due to moisture absorption by the material. The tensile speed was set at 1 mm/min to allow accurate measurement of the stress–strain relationship. The stress–strain data generated during the tensile test were recorded using the mechanical data recording system of the universal testing machine. Simultaneously, the resistance variation of the composite material during the tensile process was recorded by the digital bridge, enabling synchronized analysis with the mechanical data. Each type of sensor was prepared in five replicates, with ten repeated measurements taken for each sample. The average values were used for evaluation and inference, ensuring the reliability and accuracy of the results. The FT-IR spectra were collected within the 500~4000 cm^−1^ range using attenuated total reflection (ATR) mode on a Fourier transform infrared spectrometer (FT-IR, NICOLET 5700 FT-IR Spectrometer, Thermo Fisher Scientific, Waltham, MA, USA). X-ray photoelectron spectroscopy (XPS, PHI5000 VersaprobeI, ULVAC PHI, Tokyo, Japan) was used to characterize the sample. X-ray diffraction (XRD, Empyrean, Malvern Panalytical, Almelo, The Netherlands) (scanning angle range: 5~90°, scanning speed: 5°/min) was used to analyze the crystal structure of the sample. The sensors were also mounted on different body parts using transparent, breathable medical tape to detect human movement and physical signals.

The finite element analysis of the uniaxial stretching of fibers was conducted using the commercial software Abaqus. The fiber cross-section is circular. Regarding the boundary conditions, two fibers are arranged at a 90° angle to each other, aligned parallel to the X and Y axes. Displacement boundary conditions are applied to the cross-sections at both ends of the fibers, restricting all degrees of freedom except for the axial direction, allowing the fiber to stretch only along its axial direction. A constant displacement rate (v = 10 mm/s) is applied along the axial direction of the fiber cross-sections at both ends to simulate the stretching load. The displacement rate is equal in magnitude and opposite in direction at both ends of the same fiber. The analysis step type is selected as the ABAQUS/Explicit module to simulate the deformation process during stretching. The finite element model is based on continuous elements, and the fibers are connected through Tie contact, ensuring that their surfaces remain bonded throughout the process. Since large deformations are expected during stretching, the NLGEOM option is activated in the analysis step settings. To improve computational efficiency, mass scaling is applied in the analysis step. This method artificially increases the material density to accelerate the calculation process. In this study, a mass scaling factor of 100 is applied throughout the analysis steps.

Air permeability test: Vaseline was used to cover different substrates on a bottle containing deionized water (1 g), and then, the experiments were carried out at a temperature of 25 °C and a humidity of 50%. The mass of water remaining in the bottle was weighed every 24 h to indicate air permeability.

Ethical statement: Experiments involving human subjects were performed with the full informed consent of the volunteers. All reported tests meet the ethical requirements of the Hubei University of Technology.

## 3. Results

### 3.1. Design and Fabrication of Tri-LICN M/C/A@T Composite Sensors

To address the challenges faced by wearable applications that require stretchable strain sensors capable of accurately and stably measuring large tensile strains on the human body, three key areas were focused on: the structural design of the flexible substrate, the selection of conductive materials, and the design of the conductive network. As illustrated in the schematic diagram of the fabrication process (Figure 2a), a TPU fiber membrane was prepared as the flexible substrate through electrospinning. This method enhances the sensor’s deformation capability by utilizing TPU’s high elongation at break, flexibility, and additional advantageous properties. To further extend the strain range of the sensor, inspiration was drawn from the buckled structures observed in nature, ranging from the macroscale to the microscale and even nanoscale [35]. The TPU fiber membranes were designed with buckled structures to improve the stretchability of the flexible substrate. These buckled structures were fabricated through a hot ethanol solvent swelling treatment.

MXene was selected as the conductive material for its advantageous properties. To mitigate the dense stacking of 2D MXene sheets, our strategy involves introducing conductive materials of varying dimensions and designing a cooperative sensing network using multivariate hybrid composites. CNC was combined with MXene to leverage electrostatic interactions, stabilizing MXene dispersion in water and preventing sheet aggregation. Simultaneously, hydrogen bonding was utilized to promote the effective incorporation of MXene onto the TPU substrate. However, CNC is electrically insulating, which presents a new challenge: the electron transfer between MXene sheets is hindered by the insulating CNC, resulting in reduced electrical performance and sensitivity of the sensing network. To address this issue, highly conductive 0D materials, specifically AuNPs, were introduced to enhance energy density and establish rapid charge transfer pathways. This modification improves both the sensitivity and rapid response of the stretchable strain sensors. Additionally, AuNPs were selected for their beneficial properties, including chemical inertness, biocompatibility, and antimicrobial characteristics [25].

The conductive network was first constructed by anchoring nanoparticles onto the surface of TPU fibers using ultrasound-assisted technology. This approach prevents the detachment of MXene under large strain, thereby enhancing the stability of the stretchable sensors. The interfacial self-assembly process facilitated the uniform dispersion and anchoring of the 0D/1D/2D (AuNPs/CNC/MXene) triple-level composite onto the TPU fibers. The resulting Tri-LICN, with its multivariate self-assembled structure, enriches the conductive pathways, thereby improving both sensitivity and response time during stretching. Additionally, the synergistic effect of Tri-LICN, combined with the buckled structure of the TPU fibers, enhances the fracture toughness of the stretchable sensors, extends the sensing range, and enhances overall stability.

Additionally, flexible substrates featuring a buckled structure and self-assembled conductive networks of triple-level composites were fabricated using simple and versatile techniques, including electrospinning, hot ethanol solvent swelling treatment, and ultrasound-assisted methods. This approach offers a promising strategy for large-scale, cost-effective production. The resulting Tri-LICN M/C/A@T composite exhibits a large specific surface area, high porosity, and excellent biocompatibility. It also demonstrates resistance to sweat, breathability, thinness, and skin conformability, ensuring user comfort and compliance during prolonged wear. These properties are essential for wearable applications.

### 3.2. Structure and Morphology

**Figure 3:**
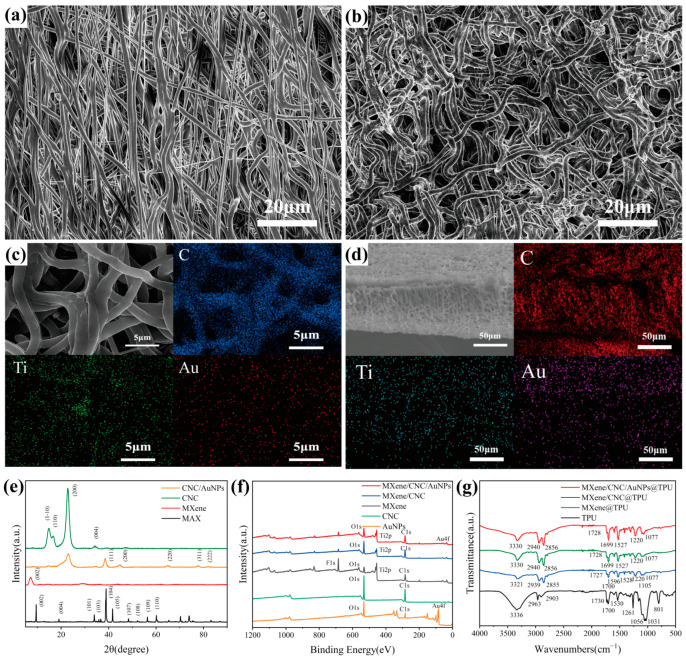
Microstructure of Tri-LICN M/C/A@T composite. SEM images of (**a**) traditional straight-TPU microfibers and (**b**) buckled-TPU microfibers. Magnification is × 1000. (**c**,**d**) are EDS mapping analysis of surface and cross-section morphology of Tri-LICN M/C/A@T composite, respectively. (**e**) XRD characterization of MAX, MXene, CNC, and CNC/AuNPs. (**f**) XPS analysis of MXene, CNC, AuNPs, MXene/CNC, and MXene/CNC/AuNPs. (**g**) FTIR spectra of TPU, TPU/MXene, TPU/MXene/CNC, and TPU/MXene/CNC/AuNPs.

Figure 3a shows SEM images of TPU fiber membranes obtained at a collector drum speed of 1000 rpm during electrospinning. The high boiling point and non-volatile DMF create physical cross-links between the fibers during the electrospinning process, thereby enhancing the mechanical robustness of the Tri-LICN M/C/A@T composite [4]. The TPU fiber membrane obtained after a hot ethanol solvent swelling treatment is shown in Figure 3b. The fibers exhibited a buckled structure with a diameter of about 1.9 μm. It was observed that the length dimensions of the TPU fiber membrane were reduced by 35% while the thickness increased. This buckled structure conferred greater elongation at break and superior elastic recovery to the TPU fiber membrane. The proposed mechanism of the hot solvent swelling process that builds the buckled structure involves the following: During the electrospinning process, tension is applied to the TPU fibers due to the high-speed rotation of the drum, causing the entangled polymer chain network of the semi-dilute solution to stretch under tension and store a certain amount of elastic energy. This process leads to a stable orientation of the polymer within the fiber. The rapid evaporation of the solvent generates a gradient coagulation field in the fiber, freezing the structure and producing a gradient pre-strain field. This process induces an unbalanced tensile conformation of the macromolecular chain [36]. When the TPU fiber membrane is immersed in ethanol at 60 °C for 1 h, this unbalanced tensile conformation of the macromolecular chain relaxes and spontaneously curls due to the dual stimulation of heating and ethanol activation, resulting in the contraction of the TPU fiber membrane and the formation of a buckled structure.

The surface and cross-section of the Tri-LICN M/C/A@T composite, obtained after ultrasound-assisted anchoring treatment, are depicted in Figure 3c,d, respectively. The EDS images reveal that MXene, CNC, and AuNPs are uniformly distributed on the fiber surface and the cross-section. This finding suggests that the triple-level M/C/A composites can be effectively dispersed and anchored within the porous and buckled fiber membranes using ultrasound-assisted anchoring, forming a complete conductive network. This phenomenon is attributed to the collapse of ultrasonic cavitation bubbles, which generate transient high temperatures, microjets, and intense shock waves, thereby facilitating the anchoring of nanoparticles on the surface of the TPU fibers [15].

Figure S1 shows the SEM images of the prepared MXene nanosheets, which are well-defined flakes with a transverse size of approximately 1–2 μm. Additionally, the MXene nanosheet suspensions exhibit a clear Tyndall effect when irradiated by a laser beam. This indicates the nanoscale, less-layered structure of the MXene nanosheets, which can be well dispersed in water [37]. From the XRD diffraction patterns of MAX and MXene in Figure 3e, it can be observed that, compared to MAX (Ti_3_AlC_2_), the diffraction peak (104) of the Al element at 2θ = 38.9° disappears in the MXene diffraction pattern, and the (002) peak shifts from 9.5° to 7.1°, confirming the successful preparation of the 2D MXene nanosheets [38]. In Figure 3e, CNC shows typical cellulose I crystallization peaks with characteristic peaks at (1–10), (110), (200), and (004) located at 14.8°, 16.6°, 22.7°, and 34.4°, respectively, indicating the successful preparation of CNC [22]. The CNC/AuNP sample displays new peaks at 38.6°, 44.7°, 64.6°, 77.8°, and 81.7°, corresponding to the (111), (200), (220), (311), and (222) phases of the face-centered cubic (FCC) crystal structure of AuNPs, respectively, confirming the successful preparation of AuNPs. These peaks are consistent with the FCC crystal structure of gold (JCPDS 04-0784), while the main XRD peaks of CNC remain intact. Interestingly, the M/C1.5 suspension in Figure S2 demonstrated superior stability after one month of storage compared to other MXene: CNC mass ratios, exhibiting the Tyndall effect. This stability is attributed to the fact that CNC, with its surface hydrophilic hydroxyl (-OH) groups and hydrophobic, highly crystalline nuclei, maintains good dispersion stability in water. The electrostatic interactions between CNC and MXene contribute to the stable dispersion of MXene in water and prevent the aggregation of MXene nanosheets [18].

The chemical structures of MXene, CNC, AuNPs, MXene/CNC, and MXene/CNC/AuNPs composites were investigated using XPS, as shown in Figure 3f. The peaks appearing at 284 eV, 455 eV, 531 eV, and 685 eV in the XPS spectra of MXene correspond to the C 1s, Ti 2p, O 1s, and F 1s characteristic peaks, respectively [37]. These Ti_3_C_2_T_X_ peaks were detected in all MXene-containing samples, indicating that the structure of MXene remained intact during the mixing process. After the introduction of CNC, the F 1s and Ti 2p signal peaks in the MXene/CNC spectra were noticeably weakened, likely due to hydrogen bonding interactions between the hydroxyl groups on the CNC surface and the -OH, -F, and -O groups on the MXene surface [39]. After introducing AuNPs, in addition to the C, O, and Ti signals in the XPS spectra of MXene/CNC/AuNPs, a new peak appeared at approximately 85 eV, confirming the presence of AuNPs. The two peaks presented at 83.78 eV and 87.44 eV correspond to Au 4f_7/2_ and Au 4f_5/2_ of the inverse fold product of Au 4f, respectively [26]. This confirms that AuNPs exist solely in their metallic state, which is beneficial for maintaining electrical conductivity in the composite.

Infrared spectral analysis of the fiber membrane samples, including TPU, MXene@TPU, MXene/CNC@TPU, and MXene/CNC/AuNPs@TPU, is shown in Figure 3g. The absorption peaks near 1530 cm^−1^ and 3336 cm^−1^ in the TPU fiber membrane are attributed to the N-H stretching and bending vibrations of the ester. The peaks at 2963 cm^−1^, 1056 cm^−1^, and 1031 cm^−1^ are attributed to the C-H, C-O, and C-O-C stretching vibrations, respectively [1]. The appearance of a double peak at 1730 cm^−1^ and two strong vibrations near 1600 cm^−1^ is attributed to the -H-N-COO- vibrations [14]. It can be clearly observed that all the samples exhibit the characteristic peaks of TPU. The MXene@TPU fibrous membrane spectrum shows the typical stretching vibration peaks of -OH (3321 cm^−1^), C-H (2939 cm^−1^), and C=O (1727 cm^−1^ and 1077 cm^−1^), which are the signature bands of Ti_3_C_2_T_X_. The -OH peak of MXene@TPU overlaps with the N-H peak of the TPU fiber membrane, compared to the pure TPU fiber membrane. The C=O and N-H vibrational peaks shift slightly to lower wavelengths (1727 cm^−1^ and 3321 cm^−1^, respectively), indicating that the C=O and N-H groups interact with the functional groups of MXene through hydrogen bonding. This interaction enhances the electromechanical properties and facilitates effective dynamic tensile load transfer in the fiber membrane. After adding CNC, the peak located at 2940 cm^−1^ in the MXene/CNC@TPU fiber membrane corresponds to the stretching vibration of the -CH group in the polysaccharides. The characteristic absorption peaks at 1527 cm^−1^, 1699 cm^−1^, and 1077 cm^−1^ are attributed to the stretching vibrations of CH2, C-O, and C-O-C in CNC, respectively. Additionally, the characteristic peak of -OH shifts to a higher position at 3330 cm^−1^, which is primarily attributed to the formation of hydrogen bonds between the abundant hydrophilic functional groups (-O, -OH, -F) on the surface of MXene and the hydroxyl group (-OH) in CNC. In the MXene/CNC/AuNPs@TPU fiber membrane spectrum, the chemical composition of the mixed samples remains almost unchanged (Figure 3f). Most of the peaks related to CNC remain unchanged, indicating that adding AuNPs does not disrupt the CNC but rather disperses it uniformly.

Based on the above characterization results, the ordered-layer self-assembly mechanism of the 0D/1D/2D (AuNPs/CNC/MXene) triple-level composite sensing networks on TPU fibers can be inferred, as shown in Figure 1b. The abundant hydrophilic functional groups (-O, -OH) on the surface of MXene and the hydrophobic -F groups on MXene were utilized to facilitate the self-assembly of MXene with CNC. This was achieved through interactions between MXene’s surface functional groups (-O, -OH, -F) and CNC’s -OH groups via hydrogen bonding and van der Waals forces. The self-assembly of MXene onto TPU fibers was facilitated by hydrogen bonding interactions between the -OH groups on MXene and the C = O and N-H groups of TPU fibers [19]. The hydrophilic functional groups (-OH) on the surfaces of both CNC and MXene, along with the highly hydrophilic surface of the AuNPs, contribute to the attachment of AuNPs to the surfaces of MXene and CNC. Additionally, physical effects such as transient high temperatures, microjets, and strong shock waves generated by the collapse of cavitation bubbles during the ultrasound-assisted anchoring process further enhanced the uniform dispersion and anchoring of the MXene/CNC/AuNPs composites onto the TPU fiber membrane. In conclusion, through the ordered-layer self-assembly strategy, interactions among AuNPs, CNC, MXene, and TPU fibers were established via hydrogen bonding, electrostatic interaction, and hydrophilicity, resulting in the construction of a complete Tri-LICN M/C/A@T composite. The ultrasonic cavitation process effectively facilitated the uniform loading and solid anchoring of the composite conductive network onto the TPU fibers.

### 3.3. Mechanical Properties

Figure 5a demonstrates the tensile mechanical properties of the fiber membrane samples un-T200, un-T500, and un-T1000, which were collected at 200 rpm, 500 rpm, and 1000 rpm during electrospinning, respectively. It also shows the properties of the samples T200, T500, and T1000 after the hot solvent swelling treatment. The un-T200 sample exhibits a tensile strength of 1.57 MPa and a tensile strain of 485%. The T200 sample shows a tensile strength of 2.99 MPa and a tensile strain of 755%. For the un-T500, the tensile strength is 3.29 MPa, with a tensile strain of 346%. The T500 demonstrates a tensile strength of 5.24 MPa, with a strain of 664%. The un-T1000 has a tensile strength of 4.34 MPa and a tensile strain of 214%. Lastly, the T1000 sample presents a tensile strength of 5.6 MPa and a tensile strain of 250%. The results reveal that the tensile strength of the fiber membranes increases, while the elongation at break significantly decreases as the collection roller speed increases. This noticeable difference can be attributed to the orientation of the TPU fibers. During electrospinning, the high-speed rotation of the collector drum stretches the TPU molecular chains under tension, leading to a well-aligned polymer structure. The faster the collector drum speed, the higher the degree of fiber orientation. Since the tensile testing was performed in the circumferential direction of the collection roller, more TPU fibers aligned along the tensile direction, which helped to withstand the axial tensile force. After the hot solvent swelling treatment, the samples exhibited a buckled structure. The higher the speed of the collection roll, the greater the gradient pre-strain generated. This leads to a more significant shrinkage of the TPU fiber film induced by the hot solvent swelling treatment, resulting in more severe buckling. The shrinkage of fiber film at a low rotational speed (200 rpm) is only 15%, whereas at a high rotational speed (1000 rpm), it is 35%. This buckled structure contributes to higher elongation at break and tensile strength in the TPU fiber membranes. This behavior is attributed to the buckled structure during the initial stretching phase. Firstly, the pre-strain stored in the buckled structure is released, causing the fiber to straighten. Subsequently, the fiber undergoes deformation due to stress, and as a result, the tensile process naturally introduces an additional margin.

**Figure 4:**
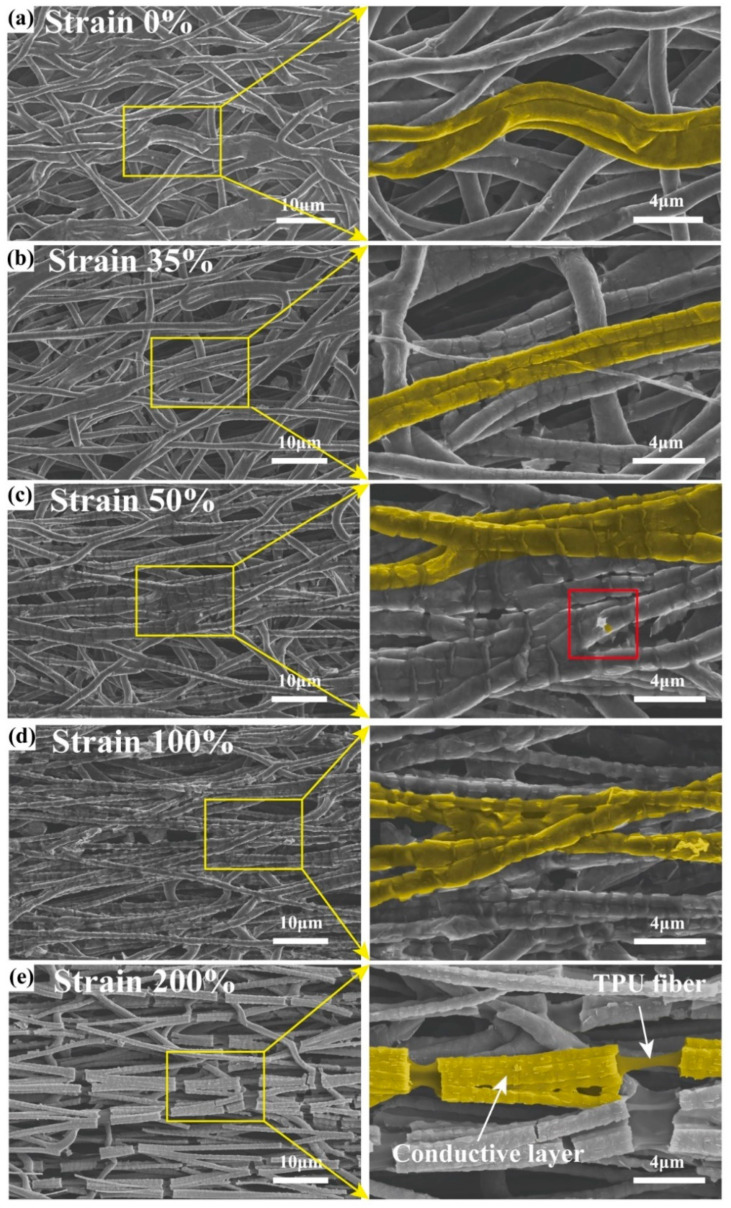
SEM images of the surface morphology for buckled-TPU microfibers under continuous mechanical stretching up to 200% strain. (**a**) strain 0%. (**b**) strain 35%. (**c**) strain 50%. (**d**) strain 100%. (**e**) strain 200%. The magnification is ×2000, with the partial enlarged detail displaying a magnification of ×6500.

**Figure 5:**
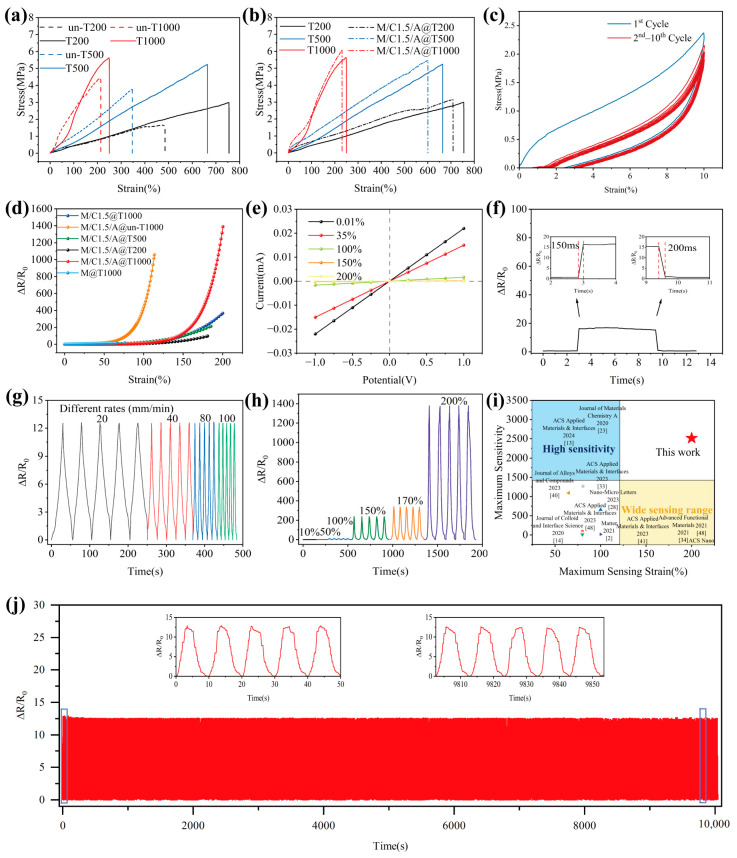
Electromechanical performance of Tri-LICN M/C/A@T composite sensor. Stress–strain curves of (**a**) traditional straight-TPU and buckled-TPU membrane and (**b**) Tri-LICN M/C/A@T composite under unidirectional tensile loading. (**c**) Cycling tensile tests for M/C1.5/A@T1000 composite. (**d**) Relative resistance variation of Tri-LICN M/C/A@T composite sensor as function of tensile strain. (**e**) I–V curves of Tri-LICN M/C/A@T composite sensor under different compressive strains. (**f**) Electromechanical response and recovery time of Tri-LICN M/C/A@T composite sensor. (**g**) Fractional resistance changes of Tri-LICN M/C/A@T composite sensor at constant strain of 100% at different stretching loading rates and (**h**) at different stretching strains with constant loading rate. (**i**) Sensitivity and sensing range comparison of Tri-LICN M/C/A@T composite sensor with state-of-the-art counterparts. (**j**) Cycling stability of M/C1.5/A@T1000 strain sensor over 1000 cycles under 100% strain.

The tensile mechanical properties of the fiber membrane samples, both before and after ultrasound-assisted anchoring treatment (Figure 5b), reveal that the tensile strength increases while the elongation at break decreases following treatment. The tensile strength of M/C1.5/A@T200 is 3.15 MPa, accompanied by a tensile strain of 700%. For M/C1.5/A@T500, the tensile strength is 5.46 MPa, with a tensile strain of 600%. The M/C1.5/A@T1000 exhibits a tensile strength of 6 MPa and a tensile strain of 230%. This enhancement in tensile strength is attributed to the ultrasound-assisted anchoring treatment, which promotes the uniform dispersion and anchoring of the triple-level M/C/A composite onto the TPU fiber membrane. The CNC between adjacent MXene nanosheets facilitates interlayer slip, thereby improving the MXene’s adaptability to interfacial inelastic deformation and providing additional frictional energy dissipation [10]. Furthermore, the MXene/CNC and MXene/TPU fibrous membranes interact through hydrogen bonding. These interactions transfer the mechanical properties of the MXene/CNC composites to the TPU fibrous membranes, thereby restricting their elongation. Consequently, greater force is required to stretch the fiber membrane in the presence of these interactions. Real-time SEM images were captured at different stages of the tensile process for Tri-LICN M/C/A@T composite to explore the tensile deformation and fracture behavior further. This was achieved by controlling the displacement of the in situ loading stage attached to the SEM, as illustrated in Figure 4a–e. In the initial state, a continuous, uniform, and complete conductive cladding layer is formed on the surface of the buckled fibers, as shown in Figure 4a. As stretching begins, the strain is partially counteracted by the gradual straightening of the fibers’ buckled structure as the tensile strain increases. When the strain reaches 35%, as shown in Figure 4b, the fibers aligned in the tensile direction are fully straightened, and the buckled structure is eliminated. This process distributes the tensile force and increases the strain range, which is a key reason for the large-strain performance of Tri-LICN M/C/A@T composite. The fiber surface cladding remained continuous, and the pores of the fiber membrane did not significantly reduce in size. As stretching continues, the fiber length increases while its diameter decreases. The MXene nanosheets are influenced by CNC between the layers, causing interlayer slip as the stress increases. Therefore, when the strain reaches 50%, a small white crack appears on the fiber surface, as shown in the red box in Figure 4c. Surrounding the crack are MXene nanosheets, with cracks between the sheets just beginning to form. The MXene nanosheets effectively transfer the load, preventing the disconnection of the surface cladding and limiting the extension of the fiber membrane. As shown in Figure 4c, the cracks in the surface cladding layer of the fibers remain continuous, despite significant widening. This indicates that the interlayer slip of the MXene nanosheets and the frictional energy dissipation of CNC prevent the disconnection of the surface cladding layer. When strain increases by 100%, as shown in Figure 4d, the cracks in the cladding layer on the fiber surface widen further, and the MXene nanosheets bridge the cracked cladding layers. The slipped and bridged MXene nanosheets effectively transfer load, withstand tensile forces, and prevent further extension of the fiber membrane, thereby enhancing its tensile strength. Additionally, the fiber diameter is reduced to 1.3–1.6 μm. Due to transverse compression perpendicular to the strain direction, the pores in the fiber membrane are flattened and elongated. As stretching continues, the hydrogen bonds between the MXene nanosheets and the TPU fiber membrane begin to break. Consequently, when the strain increases by 200%, as shown in Figure 4e, the fiber diameter further decreases. Most conductive cladding on the fiber surface breaks off, exposing the non-conductive TPU fibers.

The mechanical finite element analysis of the tensile process for un-T1000 and T1000 fibers (Figure 1d) confirms the above behavioral pattern. Under tensile loading, the stress distribution on the T1000 fibers follows a cyclic gradient in line with their cyclic buckled structure. Localized stress concentrations are observed at the apex of the semicircular region in the cyclic buckling unit, where the most pronounced deformation occurs. In contrast, the simulation results for un-T1000 fibers show a uniform stress gradient along the entire fiber. The localized stress concentrations are the primary cause of the disconnection of the conductive cladding layer on the fiber surface, as shown in Figure 4e. Furthermore, the pre-strain stored in the buckled structure of the T1000 fiber provides sufficient deformation redundancy during initial stretching, enabling them to withstand tensile strains up to 250% before failure. The un-T1000 fiber, with a lower elongation capacity, fractures when the strain reaches 200%. Based on the buckled behavior of the fibers, the Tri-LICN M/C/A@T composite can accommodate a considerably wider strain range while maintaining high sensitivity when straightened.

Figure 5c demonstrates the mechanical properties of the Tri-LICN M/C/A@T composite sample during cyclic tensile loading and unloading. A stress–strain hysteresis loop curve, representing dissipated energy, was observed during the first stretching cycle, which coincides with the fracture of the conductive cladding layer shown in Figure 4e. It indicates that the deformation of the sample structure during the initial tensile cycle involves both elastic and irreversible plastic deformation. In subsequent cycles, the hysteresis loop curves are significantly smaller than in the first cycle and nearly overlap after three cycles, indicating stabilization of the destruction and reconstruction of the conductive network. This observation is consistent with the electromechanical behavior. Therefore, it is anticipated that highly stable and reproducible sensors can be achieved after only a few cycles of mechanical training. Since TPU is a thermoplastic material, both elastic and plastic deformation occur during tensile loading. Upon unloading the tensile stress, elastic deformation recovers rapidly, while plastic deformation remains, resulting in mechanical hysteresis [40]. The area between the loading and unloading curves represents the energy lost during each cycle. This energy loss primarily stems from the work required to overcome friction within the triple-level M/C/A composite, such as the dissipation of frictional energy during interlayer sliding of the MXene. During this process, the TPU fiber membrane substrate, due to its excellent elasticity, causes the Tri-LICN M/C/A@T composite to shrink. Consequently, the triple-level M/C/A composite anchored to the membrane returns to its original position, and the microcracks revert to their initial state. This contributes to the durability and long-term stability of the stretchable sensor. No AuNPs, CNC, or MXene were shed during cyclic stretching. Furthermore, the Tri-LICN M/C/A@T composite has a thickness of approximately 110 μm, making it easily bendable and foldable, as shown in Figure 2a. As a result, the sensor exhibits excellent tensile properties and a low modulus, which enhances its potential for use in smart wearable devices with a wide monitoring range.

Consequently, this buckled structure imparts higher elongation at break and excellent elastic recovery to the TPU fiber membrane. The triple-level M/C/A composites anchored to the surface of TPU fiber membranes interact with each other through various mechanisms, including sliding and bridging. These interactions enable the membrane to withstand higher levels of strain. This deformation pattern allows for large tensile deformations while minimizing local tensile stresses, effectively facilitating the self-healing of tensile damage.

### 3.4. Strain Sensing Performance

The sensitivity of the sensor is quantified by the gauge factor (GF), defined as the ratio of the relative change in resistance to the applied strain, as given by the following equation [41]: *GF* = (∆*R*/*R*_0_)/*ε*, ∆*R* = |*R* − *R*_0_|, where *R*_0_ is the initial resistance, *R* is the resistance under applied strain, and *ε* represents the strain. Figure 5d illustrates the variation in the relative resistance of the sensor samples during stretching, with the slope of the curve indicating GF. The M@T1000 sensor displays the lowest sensing performance, while the Tri-LICN M/C/A@T composite sensor exhibits the highest overall performance, providing an optimal operating range and sensitivity. As shown in Figure 5b, although the Tri-LICN M/C1.5/A@T1000 composite has the lowest elongation at break, all other sensors failed before reaching 200% strain. Therefore, the Tri-LICN M/C1.5/A@T1000 composite sensor was selected for further studies on sensing performance and application validation. The relative resistance change curve of the Tri-LICN M/C/A@T composite sensor was divided into four regions: 0–35%, 35–100%, 100–150%, and 150–200%, with corresponding sensitivities of 1.43, 18.46, 239.58, and 2514, respectively. Figure 2b illustrates the mechanism of strain sensing during stretching, encompassing bending deformation, the sliding effect, the tunneling effect, and the jumping effect. These mechanisms correspond to the four regions of the relative resistance change curve, which are analyzed and explained in four stages. In the initial state (Figure 4a), the Tri-LICN M/C/A composites are well bonded to the buckled-TPU fiber membrane with a uniform and continuous distribution, and the conductive network has a high contact area and electron transfer efficiency. The highly conductive AuNPs facilitate rapid charge transfer, resulting in a lower initial resistance, *R*_0_ [42]. In the first stage (strain 0–35%), the buckled fiber structure shown in Figure 2b straightens along the tensile direction, partially counteracting the applied strain. Although the sensing material cladding on the fiber surface remains continuous and the sensing network is unaffected, the sensing material anchored to the fiber moves with it [43], slightly altering the relative positions within the continuous conductive network. Additionally, some contact points between the fibers shift as the buckled fibers elongate, causing a few conductive paths to be lost. Consequently, the total resistance changes gradually. Macroscopically, the fiber length increases while its cross-sectional area decreases. According to the resistance formula [44]: *R* = *ρL/A*. where *ρ* is the resistivity, *L* is the length, and *A* is the cross-sectional area; the resistance increases with increasing tension as the overall length (*L*) of the fiber membrane sensor increases and its cross-sectional area (*A*) decreases. In the second stage (35–100% strain), as shown in Figure 2b “Sliding effect” [7], the 2D MXene nanosheets begin to slip between layers along the stretching direction due to the cohesive interactions with CNC. The neighboring MXene layers remain overlapped, ensuring a strong connection within the conductive network. Additionally, the 1D CNC acts as a “bridge”, connecting the neighboring MXene sheets, efficiently transferring electrons together with AuNPs. As more MXene sheets begin to slip and the overlap area between the slipping MXenes decreases, the total resistance of the sensor increases gradually while the sensitivity also rises slowly. In the third stage (100–150% strain), nanoscale gaps appear between the conducting materials in the sensing network. As shown in Figure 2b, according to the “tunneling effect” described by Simmons, electrons can move across these nanoscale gaps between the conducting particles [45]. The enhancement of the tunneling effect has a lesser impact on electron mobility than the reduction in the contact area between the MXene sheets. Therefore, the combined effects of sliding and tunneling accelerate the increase in total resistance and the enhancement of sensitivity. In the fourth stage (150–200% strain), the distance between the conducting particles exceeds the potential barrier that electrons can easily overcome, leading to the gradual failure of the tunneling effect. Some electrons are still able to jump within a variable range, referred to as the “jump effect” [46], as shown in Figure 2b. As a result, the total resistance of the sensor changes dramatically, and the sensitivity increases rapidly. When the strain exceeds 200%, a large number of cladding layers of the sensing material on the fiber surface are disconnected, exposing the non-conductive TPU fibers. This leads to the formation of visible and irreversible cracks on the surface of the Tri-LICN M/C/A@T composite membrane (Figure 4e). Therefore, the optimal operating range for the Tri-LICN M/C/A@T composite sensor is 200%. In contrast, the strain sensing range of the M@T1000 sensor is limited to 0–70% (Figure 5d). This limitation is attributed to the stacking of MXene sheets on the TPU fiber membrane, which causes uneven load distribution. As a result, cracks rapidly propagate, disrupting the connections between MXene sheets and leading to premature failure of the conductive pathways. The maximum sensitivity of the Tri-LICN M/C1.5@T1000 composite sensor is only 532.83, which is only one-quarter of the sensitivity of the Tri-LICN M/C1.5/A@T1000 composite sensor. This indicates that AuNPs enhance the electronic transport efficiency of the sensor network, thereby improving the sensor’s sensitivity. The range of the Tri-LICN M/C1.5/A@un-T1000 composite sensor is significantly lower than that of the Tri-LICN M/C1.5/A@T1000 composite sensor, indicating that the buckling structure increases the deformation of the composite, thereby enhancing the strain range of the sensor. The sensitivity of the Tri-LICN M/C1.5/A@T200 and M/C1.5/A@T500 composite sensors is also much lower than that of Tri-LICN M/C1.5/A@T1000 composite sensors. This discrepancy can be attributed to the TPU fiber diameter variations and the interwoven overlapping structure, which constructs different scaffold-like network structures based on the stress field of the TPU substrates. Consequently, the conductive paths formed by the embedded conductive fillers exhibit significant differences, resulting in varying macroscopic response sensitivities [45]. When electrospun TPU fibers are collected at higher spinning speeds, they align more orderly, and the resulting conductive paths become more tightly packed, resulting in increased sensitivity. In conclusion, the Tri-LICN M/C/A@T composite sensor exhibits stable sensing performance with high sensitivity over a broader working range, benefiting from the unique electromechanical properties of the 0D/1D/2D(AuNPs/CNC/MXene) triple-level composite and the synergistic effects of buckled structures and multiple mechanisms under different strain conditions.

The rapid response of the strain sensor is beneficial for the real-time monitoring of fast and complex motions. In Figure 5f, the response time and recovery time of the Tri-LICN M/C/A@T composite sensor are ~150 ms and ~200 ms, respectively (Figure 5g), indicating its suitability for high-frequency monitoring. The fast response capability of the strain sensor is attributed to the efficient charge transport pathways provided by the highly conductive AuNPs in the sensor network. Figure 5e presents the current-voltage (I–V) curve of Tri-LICN M/C/A@T composite sensor under 0–200% strain. The I–V curve strictly follows Ohm’s law [47], with the slope decreasing as the strain increases from 0.01% to 200%. This behavior indicates the excellent stability of the Tri-LICN M/C/A@T composite sensor under static tensile strain. The strain sensor exhibits excellent signal repeatability of ΔR/R0 under different strains or strain rates, as shown in Figure 5g,h. Cycling performance is a key parameter for evaluating the long-term robustness of strain sensors. Dynamic durability tests of the Tri-LICN M/C/A@T composite sensor were conducted under 100% large strain cycling at a rate of 50 mm min^−1^ (Figure 5j). In the initial cycles, the relative resistance response shows transient behavior due to mechanical hysteresis and changes in the conductive network. As the stretching cycles increase, the relative resistance response gradually stabilizes. This sensor can endure up to 100% strain with repeatable loading/unloading cycles up to 1000 times, with negligible performance degradation, demonstrating exceptional long-term robustness, durability, and recoverability. This is attributed to the synergy between ultrasound cavitation and hydrogen bonding, which enables the triple-level M/C/A composite to form a stable conductive network anchored firmly onto the TPU membrane, ensuring that a stable conductive network is maintained even under large strain without the conductive material detaching. The buckled-TPU fiber membrane not only withstands higher levels of strain but also drives the sensing material anchored to return to its initial position and state after strain release, thereby imparting long-term stability to the stretchable sensor under large strains. To assess the air permeability of the composite, four control experiments were performed using bottles containing 1.0 g of DI water. In these experiments, the bottle mouths were uncovered; the cap, PDMS square sheet (2 cm × 2 cm × 0.5 cm), and Tri-LICN M/C/A@T composite were covered. Daily measurements of the water content were recorded. As depicted in Figure S3, the water content in the bottle covered with the Tri-LICN M/C/A@T composite material decreased by 80%, demonstrating that the micro-nano porous structure inherent in the TPU film, prepared via electrospinning, imparted excellent air permeability to the composite. Additionally, the sensor exhibited lightweight characteristics, with a sample mass of ~0.1 g. Due to its exceptional stretchability and tensile strength (6 MPa), the composite is highly adaptable, comfortable, and soft when in contact with the skin. Compared to other recently reported sensors, the Tri-LICN M/C/A@T composite sensor exhibits significant advantages in terms of sensing range and sensitivity, as shown in Figure 5i. Compared to the strain sensors developed by Wang et al. using a template printing method to pattern liquid metal onto electrospun TPU membranes (GF ~ 2.69) and the highly breathable, pressure- and bending-insensitive strain sensor reported by Liu et al. (GF ~ 49.5), the Tri-LICN M/C/A@T composite material sensor exhibits significantly higher sensitivity [34,48]. Cui et al. achieved excellent sensitivity (GF ~ 1265.18) by vacuum filtering and self-assembling conductive fillers into polymer porous films with asymmetric structures [33]. However, the sensor’s sensing range is only 80.7% of that of the Tri-LICN M/C/A@T sensor, which is merely 2/5 of its range. These comparisons underscore the superior performance of the Tri-LICN M/C/A@T sensor, making it a promising candidate for ultra-stretchable strain sensors. A thorough review and analysis of the existing literature reveals the existing imbalance between the sensing range and sensitivity of sensors. By addressing the stretchability of the polymer substrate and the extensibility of the conductive materials, an ultra-stretchable strain sensor was developed, offering a broad sensing range and high sensitivity.

### 3.5. Applications: Physiological Perception Performance

**Figure 6:**
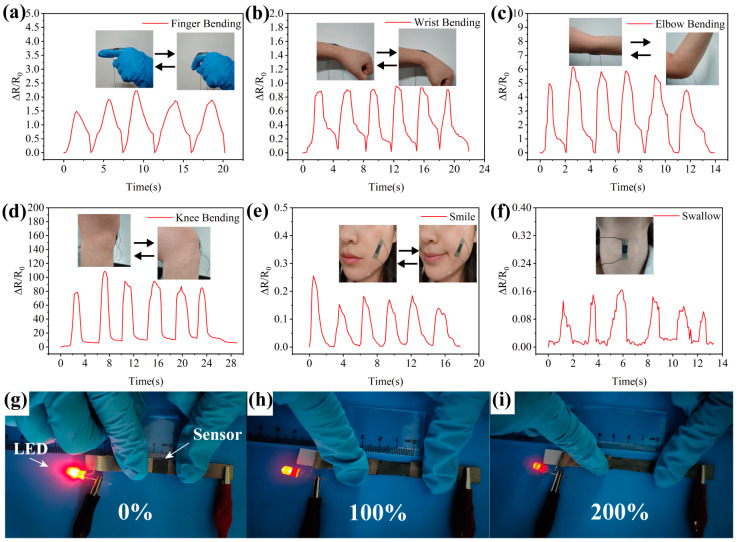
Application of our proposed ultra-stretchable Tri-LICN M/C/A@T composite sensor in human monitoring. Real-time recording of (**a**) finger bending, (**b**) wrist bending, (**c**) elbow bending, (**d**) knee bending, (**e**) facial expression—smile and (**f**) swallow. (**g**–**i**) Optical images of LED brightness by sensor under different stretching strains to display electromechanical responses of Tri-LICN M/C/A@T composite sensor.

To demonstrate the feasibility of the Tri-LICN M/C/A@T composite sensor for practical wearable health monitoring, the sensor was employed to detect human activities involving varying degrees of strain [2]. As the sensor is stretched and deformed during the movement of the human body, the displacement and reorganization of the conductive paths within the polymer matrix occur. This deformation causes a change in the material’s electrical conductivity, leading to a measurable variation in resistance [49]. To assess its capability in detecting large strains, the sensor was placed on the wrist, elbow, and knee, as shown in Figure 6a–d. The measurement results confirm that the sensor reliably distinguishes between different strain levels. During physical activity, the strain sensor effectively identified varying resistance signals, confirming its ability to differentiate between different types of motion. Based on these data, distinct physical states of the body can be differentiated based on frequency and amplitude. As shown in Figure 6h,i, the sensor exhibits responsive behavior under large strains. When the Tri-LICN M/C/A@T composite sensor was connected to an LED light powered by a 10 V source, its electrical response to various strains was evaluated. The LED’s brightness gradually dimmed as external strain increased from 0% to 200%. The current continued to decrease with further strain, reflecting the broad strain range and the sensor’s high sensitivity to conductivity changes. The sensor was attached to the skin near the corners of the mouth to monitor facial expressions. When the mouth corners were raised, the sensor was stretched to accommodate the deformation of the skin, resulting in a change in sensor resistance (Figure 6e). The Tri-LICN M/C/A@T composite sensor was placed on the vocal cords to demonstrate the sensor’s ability to detect small strains. During swallowing, the skin in the throat region experienced stretching strains ranging from 5% to 20%, resulting in a resistance change ranging from 0.01 to 0.16. In the experimental application testing, the Tri-LICN M/C/A@T composite was applied onto human skin for three days, during which no signs of redness, swelling, allergic reactions, or other adverse effects were observed, indicating favorable skin compatibility. Therefore, the wide detection range and high sensitivity of the Tri-LICN M/C/A@T composite sensor make it suitable for a variety of scenarios, demonstrating its potential as a wearable health monitoring device [48,50].

## 4. Conclusions

An ultra-stretchable composite sensor with an extensive strain detection range was developed by introducing a novel design strategy of a triple-level interaction conductive network (Tri-LICN) and buckled microfiber. The composite sensor was fabricated by anchoring a Tri-LICN consisting of 0D AuNPs, 1D CNC, and 2D MXene components onto highly buckled TPU microfiber substrates through interface self-assembly, ultrasound-assisted anchoring, and hot solvent swelling processes. The buckled microstructures of TPU microfibers enhance the deformation of the flexible substrate, while the 1D CNC bridges the 2D MXene and 0D AuNPs, forming a stable Tri-LICN. Tri-LICN significantly enhances conduction pathways and improves the modulus matching between the conductive materials and the flexible substrate. In combination with the buckled elastic structure, this network provides a synergistic enhancement, resulting in a broad operating range (0.05–200%) and excellent durability (>1000 cycles). Additionally, the sensor exhibits high sensitivity (GF ~ 2514), fast response time (~150 ms), skin affinity, breathability, lightweight properties (sample quality ~0.1 g), and skin conformability (tensile strength 6 MPa), demonstrating its potential for applications in human monitoring. This study introduces an innovative and straightforward design strategy that integrates a complex Tri-LICN, constructed from multidimensional materials, with a buckled-TPU fiber film achieved through a hot solvent swelling process. The structural design of the polymer flexible substrate is optimized to enhance its stretchability and tensile strength. Furthermore, electrostatic self-assembly is employed to form a stable conductive network consisting of MXene, CNC, and AuNPs, effectively addressing the issue of limited extensibility in the conductive network. This approach aims to improve the electromechanical performance of polymer film strain sensors. While the sensor developed in this study effectively covers a range of strain levels across various parts of the human body, its operational range currently does not reach the stretching limits of the polymer substrate. Consequently, future work will focus on advancing research to further enhance sensor performance based on polymers.

## Data Availability

Data are contained within the article.

## Supplementary Materials

The following supporting information can be downloaded at https://www.mdpi.com/article/10.3390/polym17060734/s1, Figure S1: SEM image of MXene nanosheets (magnification is ×8000) and photo of MXene solution irradiated by laser; Figure S2: Pictures of different proportions of CNC dispersed in aqueous solution of MXene for one month. (a) MXene, (b) M/C0.5, (c) M/C1.0, (d) M/C1.5, (e) M/C2.0; Figure S3: Change curve of permeability of Tri-LICN M/C/A@T composite with time; Table S1: A comparison of the sensing performance of reported sensors.
