# Supplementary material for: Ultra-Stretchable Polymer Fibers Anchored with a Triple-Level Self-Assembled Conductive Network for Wide-Range Strain Detection
Source: Polymers (Basel). 2025 Mar 11;17(6):734. doi: 10.3390/polym17060734 (PMC11945324; doi:10.3390/polym17060734)
Supplement: Supplementary file 1 [file polymers-17-00734-s001.zip › Supporting Information.pdf]

Supporting Information

Ultra-Stretchable Polymer Fibers Anchored with a Triple-Level Self-Assembled Conductive Network for Wide-Range Strain Detection

Zhong Zheng [1,*], Shuyi Song [1], Xun Chen [1], Xixing Li [1] and Jing Li [1,2,*]

[1] Hubei Key Laboratory of Modern Manufacturing Quantity Engineering, School of Mechanical Engineering, Hubei University of Technology, Wuhan 430068, Hubei, China; songshuyi1016@163.com (S.S.); cx747699@163.com (X.C.); li_xi_xing@126.com (X.L.)
[2] School of Intelligent Manufacturing, Hubei University, Wuhan 430062, China
* Correspondence: zhengzh@hbut.edu.cn (Z.Z.); lijing@hbut.edu.cn (J.L.); Tel.: +86-027-59750012 (Z.Z. & J.L.)

1. SEM image of MXene and photo of MXene solution irradiated by laser.

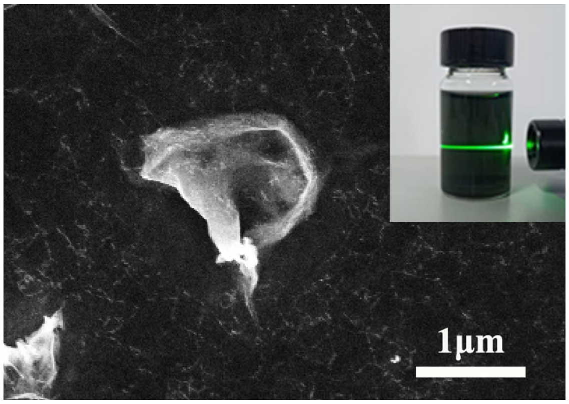

Figure S1. SEM image of MXene nanosheets (The magnification is × 8000) and photo of MXene solution irradiated by laser.

Academic Editor(s): Jem-Kun Chen

Received: 13 February 2025
Revised: 3 March 2025
Accepted: 7 March 2025
Published: date

Citation: Zheng, Z.; Song, S.; Chen, X.; Li, X.; Li, J. Ultra-Stretchable Polymer Fibers Anchored with a Triple-Level Self-Assembled Conductive Network for Wide-Range Strain Detection. *Polymers* **2025**, *17*, x. https://doi.org/10.3390/xxxxx

Copyright: © 2025 by the authors. Submitted for possible open access publication under the terms and conditions of the Creative Commons Attribution (CC BY) license (https://creativecommons.org/licenses/by/4.0/).

2. Pictures of different proportions of CNC dispersed in aqueous solution of MXene for one month

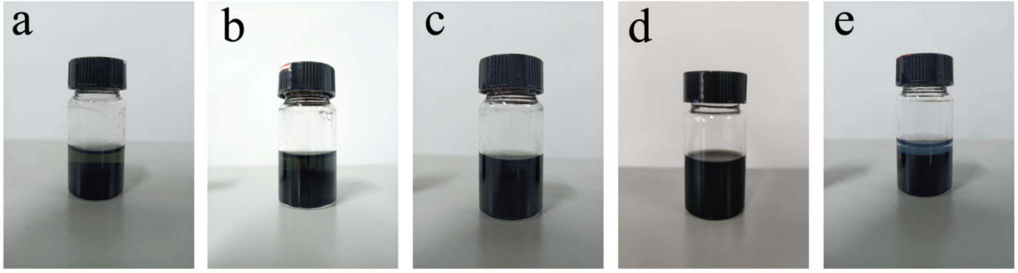

Figure S2. Pictures of different proportions of CNC dispersed in aqueous solution of MXene for one month (a) MXene (b) M/C0.5 (c) M/C1.0 (d) M/C1.5 (e) M/C2.0.

As can be seen from the Figure S2, when the mass ratio of MXene:CNC is 1:1.5, the dispersibility and stability of MXene solution are better.

3. Change curve of permeability of Tri-LICN M/C/A@T composite with time

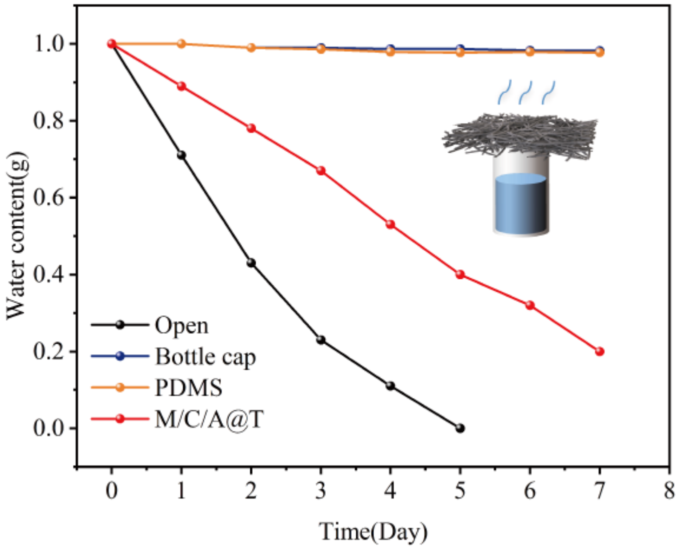

Figure S3. Change curve of permeability of Tri-LICN M/C/A@T composite with time

4. A comparison of the sensing performance of reported sensors

Table S1 A comparison of the sensing performance of reported sensors[1-11]

Ref.	Maximum sensing range (%)	Maximum Sensitivity	Response/relaxation time (ms)	Durability (cycles)
This work	200	2514	150/200	1000
[1]	100	15.61	186/-	1000
[2]	100	2080.9	-	2500
[3]	80	9.69	140.6/-	1750
[4]	83	3405	75/60	1000
[5]	100	657.36	-	1000
[6]	80.7	1265.18	66/-	2300

[7]	200	49.5	50/-	3000
[8]	200	45.34	180/180	2000
[9]	184	58.903	58.82/117.64	2000
[10]	80	90	52/85	2000
[11]	200	2.69	-	1000

References

1. H. Liu; S. Zhang; Z. Li; T. J. Lu; H. Lin; Y. Zhu;S. Ahadian; S. Emaminejad; M. R. Dokmeci; F. Xu; A. Khademhosseini. Harnessing the wide-range strain sensitivity of bilayered PEDOT:PSS films for wearable health monitoring. *Matter* **2021**, *4*, 2886-2901.
2. K. Pan; J. Wang; Y. Li; X. Lu;D. Hu; Z. Jia; J. Lin. Sandwich-Like Flexible Breathable Strain Sensor with Tunable Thermal Regulation Capability for Human Motion Monitoring. *ACS Applied Materials & Interfaces* **2024**, *16*, 10633-10645.
3. Z. Jia; Z. Li;S. Ma; W. Zhang; Y. Chen; Y. Luo;D. Jia; B. Zhong; J. M. Razal; X. Wang; L. Kong. Constructing conductive titanium carbide nanosheet (MXene) network on polyurethane/polyacrylonitrile fibre framework for flexible strain sensor. *Journal of Colloid and Interface Science* **2021**, *584*, 1-10.
4. Q. Li; R. Yin;D. Zhang; H. Liu; X. Chen; Y. Zheng; Z. Guo; C. Liu; C. Shen. Flexible conductive MXene/cellulose nanocrystal coated nonwoven fabrics for tunable wearable strain/pressure sensors. *Journal of Materials Chemistry A* **2020**, *8*, 21131-21141.
5. Q. Wang; Z. Yao; C. Zhang; H. Song; H. Ding; B. Li;S. Niu; X. Huang; C. Chen; Z. Han; L. Ren. A Selective-Response Hypersensitive Bio-Inspired Strain Sensor Enabled by Hysteresis Effect and Parallel Through-Slits Structures. *Nano-Micro Letters* **2023**, *16*, 26.
6. X. Cui; C. Miao;S. Lu; X. Liu; Y. Yang; J. Sun. Strain Sensors Made of MXene, CNTs, and TPU/PSF Asymmetric Structure Films with Large Tensile Recovery and Applied in Human Health Monitoring. *ACS Applied Materials & Interfaces* **2023**, *15*, 59655-59670.
7. Z. Liu; Y. Zheng; L. Jin; K. Chen; H. Zhai; Q. Huang; Z. Chen; Y. Yi; M. Umar; L. Xu; G. Li; Q. Song; P. Yue; Y. Li; Z. Zheng. Highly Breathable and Stretchable Strain Sensors with Insensitive Response to Pressure and Bending. *Advanced Functional Materials* **2021**, *31*, 2007622.
8. W. Liu; C. Xue; X. Long; Y. Ren; Z. Chen; W. Zhang. Highly flexible and multifunctional CNTs/TPU fiber strain sensor formed in one-step via wet spinning. *Journal of Alloys and Compounds* **2023**, *948*, 169641.
9. J. Ai; Q. Wang; Z. Li;D. Lu;S. Liao; Y. Qiu; X. Xia; Q. Wei. Highly Stretchable and Fluorescent Visualizable Thermoplastic Polyurethane/Tetraphenylethylene Plied Yarn Strain Sensor with Heterogeneous and Cracked Structure for Human Health Monitoring. *ACS Applied Materials & Interfaces* **2023**, *16*, 1428-1438.
10. G. Yang; H. Luo; Y. Ding; J. Yang; Y. Li; C. Ma; J. Yan; X. Zhuang. Hierarchically Structured Carbon Nanofiber-Enabled Skin-Like Strain Sensors with Full-Range Human Motion Monitoring and Autonomous Self-Healing Capability. *ACS Applied Materials & Interfaces* **2023**, *15*, 7380-7391.
11. M. Wang; C. Ma; P. C. Uzabakiriho; X. Chen; Z. Chen; Y. Cheng; Z. Wang; G. Zhao. Stencil Printing of Liquid Metal upon Electrospun Nanofibers Enables High-Performance Flexible Electronics. *ACS Nano* **2021**, *15*, 19364-19376.
